# Terahertz Spin‐Conjugate Symmetry Breaking for Nonreciprocal Chirality and One‐Way Transmission Based on Magneto‐Optical Moiré Metasurface

**DOI:** 10.1002/advs.202204916

**Published:** 2022-11-14

**Authors:** Zhiyu Tan, Fei Fan, Shengnan Guan, Hao Wang, Dan Zhao, Yunyun Ji, Shengjiang Chang

**Affiliations:** ^1^ Institute of Modern Optics Nankai University Tianjin Key Laboratory of Micro‐Scale Optical Information Science and Technology Tianjin 300350 P. R. China; ^2^ Tianjin Key Laboratory of Optoelectronic Sensor and Sensing Network Technology Tianjin 300350 P. R. China

**Keywords:** chirality, magneto‐optics, moiré metasurface, nonreciprocity, terahertz

## Abstract

In this work, the gyrotropic semiconductor InSb into the twisted bilayer metasurface to form a magneto‐optical moiré metasurface is introduced. Through the theoretical analysis, the “moiré angle” is developed in which case the nonreciprocity and chirality with the spin‐conjugate asymmetric transmission are obtained due to the simultaneous breaking of both time‐reversal symmetry and spatial mirror symmetry. The experiments confirm that the chirality can be actively manipulated by rotating the twisted angle and the external magnetic field, realizing spin‐conjugate asymmetric transmission. Meanwhile, the two spin states also realize the nonreciprocal one‐way transmission, and their isolation spectra are also spin‐conjugate asymmetric: one is enhanced up to 48 dB, and the other's bandwidth is widened to over 730 GHz. This spin‐conjugate symmetry‐breaking effect in the MOMM brings a combination of time‐space asymmetric transmission, and it also provides a new scheme for the implementation of high‐performance THz chirality controllers and isolators.

## Graphical abstract

A magneto‐optical moiré metasurface is proposed that can actively manipulate the chiral response and nonreciprocity with the spin‐conjugated symmetry breaking. Moreover, it obtains an enhanced one‐way transmission and broadens the operating band. The attractive functionality and unique physics mechanism of this work have great potential in 3D displays, digital encoding, and optical communication.

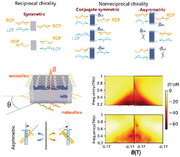

## 1 Introduction

The chirality of light is characterized by a couple of spin photons whose mirror image is not overlapping with itself, and it plays key roles in biochemical detection, drug synthesis, and mass communication technologies. For a certain object, it achieves chirality due to the structural inversion or mirror symmetry breaking, thereby obtaining a different response to chiral photons. This unique property can identify chiral objects such as molecules, medicines, and explosives.^[^ ^1^ ^]^ In communication technology, the chiral response of the photons also plays a key role in multichannel multiplexing and polarization optical systems.^[^ ^2^, ^3^, ^4^ ^]^ Therefore, the manipulation of optical chirality is especially important making chiral optics an ever‐evolving field. However, different from the UV–visible‐near‐infrared band, the chiral manipulation in the terahertz (THz, 1 THz = 10^12^ Hz) band, especially the dynamic chiral manipulation, is limited by the lack of chiral materials, which hinders the development and application of optical chirality technology in the THz band.

Magneto‐optical (MO) materials have special optical chirality under the external magnetic field (MF), such as the MO Faraday rotation (FR) effect,^[^ ^5^, ^6^, ^7^, ^8^ ^]^ MO Kerr effect,^[^ ^9^ ^]^ and magnetic circular dichroism (MCD).^[^ ^10^ ^]^ This chiral response is magnetically tunable and breaks the time‐reversal symmetry of light propagation,^[^ ^11^ ^]^ so that light transmission along the forward and reverse direction or for the forward and reverse MF is quite different to achieve unidirectional isolation, which is called nonreciprocity.^[^ ^12^ ^]^ In recent years, THz MO materials, physics, and devices have been developed, such as magneto‐plasmonics,^[^ ^13^ ^]^ magnetic photonic crystals,^[^ ^14^ ^]^ MO isolators,^[^ ^15^ ^]^ and MO modulators.^[^ ^16^, ^17^ ^]^ Among the few THz MO materials, the gyroelectric semiconductor InSb has been interested in THz MO and topological devices,^[^ ^18^, ^19^, ^20^, ^21^, ^22^, ^23^, ^24^, ^25^, ^26^ ^]^ since its cyclotron resonance is just located in the THz band under a weak external MF because of an extremely low effective mass m* = 0.014 *m*_e_. For instance, in 2018, Lin et al. reported a nonreciprocal THz reflection on the InSb with the transverse magneto‐optical effect (i.e., Voigt configuration).^[^ ^22^ ^]^ However, the device functions and performances only rely on the magnetic optical chirality or nonreciprocity of the THz MO material itself are limited. In 2019, Wang et al. found the photonic Weyl points in the topologically photonic band structures of the transverse magnetized InSb‐plasmonic grating in the THz band.^[^ ^23^ ^]^ This indicates that more novel physical mechanisms and results can be obtained by combining magneto‐optical materials with artificial micro‐nanostructures.

Recently, an intriguing structure called moiré superlattice significantly attract researchers' attention.^[^ ^27^, ^28^, ^29^, ^30^, ^31^, ^32^, ^33^, ^34^, ^35^, ^36^ ^]^ The moiré superlattice that is formed by vertically stacking two monolayers with a twisted angle is a concept in electronics, opening a new way to discover novel correlated physical fundamentals, such as superconductivity,^[^ ^27^, ^28^, ^29^ ^]^ topological physics, and moiré excitons.^[^ ^30^, ^31^, ^32^, ^33^, ^34^, ^35^, ^36^ ^]^ Due to the space inversion symmetry breaking, the moiré superlattices obtain the physical chirality (intuitively, the twisted angle *θ* is not identical to twisted angle −*θ*), observing chiral plasmons.^[^ ^36^ ^]^ Unlike the moiré lattice in electronics, due to the longer wavelength of photons and their light‐matter waves (polaritons) than that of electrons, the separation between adjacent components in twisted photonic structures can range from subnanometer to photon wavelengths.^[^ ^37^ ^]^ The moiré metasurfaces are composed of two vertically stacking metasurfaces with twisted angles. The photonic moiré metasurfaces show intriguing properties such as the topological transition of polaritonic nano‐optics,^[^ ^38^, ^39^, ^40^, ^41^, ^42^, ^43^ ^]^ chiral optics,^[^ ^44^, ^45^, ^46^ ^]^ and localization and delocalization transition of light.^[^ ^47^, ^48^ ^]^ Therefore, the photonic moiré metasurfaces are promising in tailoring the light‐matter interaction as well as manipulating the chiral light. However, in the THz band, the researches for moiré metasurfaces are still very limited.

In this work, to overcome spin‐conjugate symmetry in the nonreciprocity and chirality of bulk gyroelectric medium, we have combined the InSb with the twisted bilayer metasurfaces to form a magneto‐optical moiré metasurface (MOMM) in the THz band. A novel property can be found at the “moiré angle” that realizes the nonreciprocal chiral response with spin‐conjugate symmetry breaking. Then, we experimentally characterize this device and observe the reducing degeneracy of the conjugate spin states in the positive and negative MFs. Further, this MOMM fully utilizes the MO effect of the InSb and realizes the hyper‐broadband or enhanced nonreciprocal chirality. These mechanisms in time‐space asymmetric transmission and manipulation have promising potentials in THz fundamental physics and applications in displays, encoding, and optical communication.

## 2 Results

### 2.1 Theoretical Analysis of MOMM

**Figure 1:**
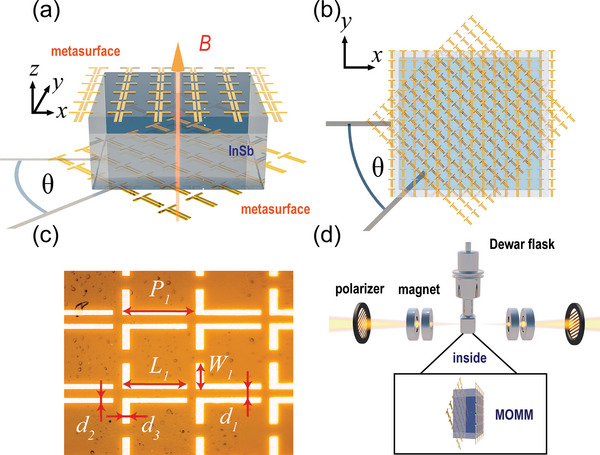
a) The sketch map of MO moiré metasurface. b) The moiré pattern of the MOMM. c) The microphotograph of the metallic metasurfaces and the geometric parameters: *P*_1_ = 80 µm, *L*_1_ = 60 µm, *W*_1_ = 30 µm, *d*_1_ = 8 µm, *d*_2_ = 10 µm, *d*_3_ = 12 µm. d) The schematic diagram of the experiment setup.

The MOMM is composed of an InSb layer sandwiched between two twisted metallic metasurfaces as shown in **Figure** **1**a. The metallic metasurfaces are rotated to a twisted angle *θ* with the InSb inserted into them, therefore forming the moiré pattern as shown in Figure 1b. The metallic metasurface is designed as a double‐L‐shaped pattern as shown in Figure 1c, which has an anisotropic response at the center frequency of 0.9 THz. The basic design principles and experimental results of the single‐layer metasurface structure can be found in Section S3 of the Supporting Information. The device fabrication can be found in the Experimental Section.

**Figure 2:**
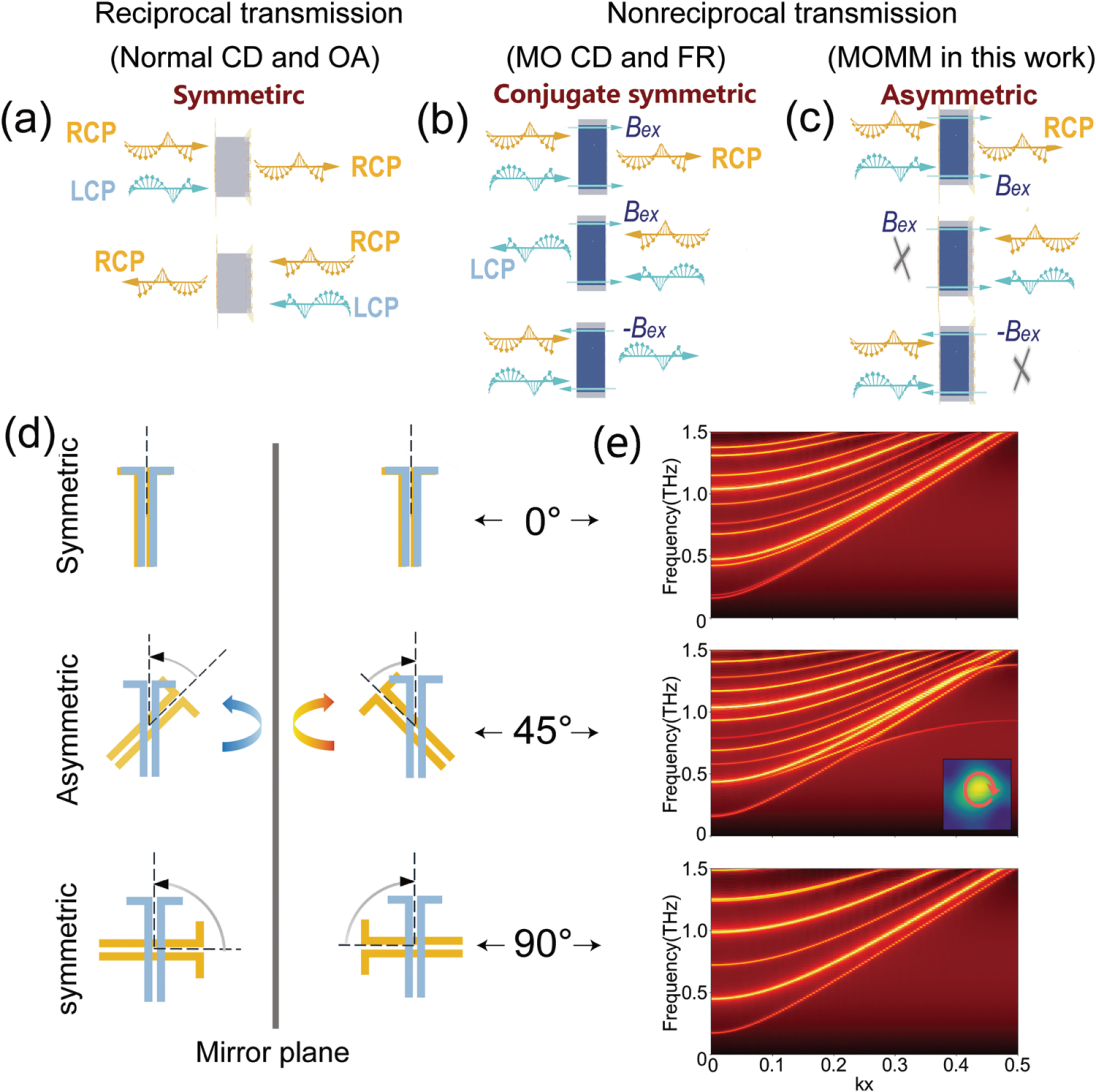
a) Schematic diagram of reciprocal chirality in moiré metasurface without InSb. b) Schematic diagram of nonreciprocal chirality in magnetized bulk InSb or MOMM with *θ* = 0°, ±90°, or 180°. c) Schematic diagram of nonreciprocal chirality with spin‐conjugate symmetry breaking in MOMM. d) The geometric symmetry of the moiré metasurfaces with different twisted angles (from top to bottom): 0°, 45°, and 90°. e) Theoretically calculated band diagram of moiré metasurfaces with the twisted angles *θ* = 0°, 45°, and 90°. For 45°, the inserted figure depicts chiral plasmons in the transmission field at 0.8 THz.

The InSb is a gyrotropic semiconductor under an external MF, exhibiting a strong cyclotron resonance effect (i.e., MCD effect) and FR effect in the THz band. In this case, we can obtain two eigen solutions of Maxwell's wave equation in the longitudinally magnetized InSb. They are a pair of the conjugate photonic spin states with right‐handed and left‐handed rotation directions (i.e., *R* and *L* states).^[^ ^26^ ^]^ The detailed theoretical analysis and experimental results for the MO properties of InSb can be found in Section S2 of the Supporting Information. Due to the time‐reversal symmetry breaking caused by the MF, the system with magnetized InSb has nonreciprocity to the spin photons. In this case, changing the direction of MF is equivalent to changing the direction of spin state transmission, so we use + and − to mark the positive and negative MF (or forward and backward transmission), respectively. A schematic diagram of spin transmission in the InSb is shown in **Figure** **2**b, and the main conclusions are as follows: 1) for the same direction of MF (or propagation), the transmission properties of the two conjugate spins are different due to the cyclotron resonance, so InSb has THz MO chirality showing the MCD effect in the cyclotron resonance band and the FR effect far from the cyclotron resonance frequency *ω*_c_. 2) For one of the spin states, its transmission characteristics are different between the positive and negative MF, showing nonreciprocal one‐way transmission. 3) Although the magnetized InSb has a nonreciprocal chirality (i.e., MCD and FR effect in fact), the two conjugate spin states are mirror‐antisymmetric to the MF as shown in Figure 2b, so we call it spin‐conjugate symmetry. All the relations discussed above are expressed as follows^[^ ^49^ ^]^

(1) $$\begin{array}{cc} & \mathrm{Optical}\;\mathrm{Chirality}:t_{R+}\neq t_{L+},t_{R-}\neq t_{L-}\\ & \mathrm{Nonreciprocity}:t_{R+}\neq t_{R-},t_{L+}\neq t_{L-}\\ & \mathrm{Conjugate}\;\mathrm{symmetry}:t_{R+}=t_{L-},t_{R-}=t_{L+} \end{array}$$

Only two of the four spin states are independent. This means that when a pair of spin states simultaneously incident into the InSb, there is always a spin state that can output, which limits the applications of the bulk InSb in spin multiplexing and one‐way transmission.

The origin of this spin‐conjugate symmetric transmission is that InSb has only the dielectric tensor asymmetry, but no spatial structure asymmetry. Therefore, we consider introducing InSb into the moiré structure with two twisted anisotropic metasurfaces to break spatial mirror symmetry. First, we discuss the case when no InSb is introduced or the MF is not applied. As shown in Figure 2d, when the twisted angle *θ* = 0°, ±90°, or 180°, the structure is mirror‐symmetric in the propagation direction, so it is achiral. When *θ* ≠ 0°, ±90°, and 180°, the spatial mirror symmetry is breaking, but the transmission system is still time‐reversal symmetric. Therefore, the transmission properties of two spins are the same when the propagation direction is reversed, and this structure indicates the normal circular dichroism or normal optical activity effect as shown in Figure 2a, that is *t*_*R* +_ ≠ *t*_*L* +_, *t*_*R* −_ ≠ *t*_*L* −_ but *t*_*R* +_ = *t*_*R* −_, *t*_*L* +_ = *t*_*L* −_. To distinguish it from the nonreciprocal MO chirality in the magnetized InSb, the chiral response of this nonmagnetic moiré metasurface is reciprocal, so it is referred to as reciprocal chirality in this paper.

We also simulate the photonic band for the moiré metasurfaces. The equivalence method is used to convert the rotating metasurface into a single periodic structure in the first Brillouin zone as shown in Figure 2e. When *θ* = 0°, the photonic band only has the transmission bands. When *θ* = 45°, the guided mode band between the two metasurface layers appears around 0.6–0.9 THz due to the symmetry breaking in the *k*‐space. The transmission field of the supercell (7×7 unit in a period) on the second metasurface can be seen in the inserted figure, which shows the chiral plasmons. Therefore, in the guided mode band, when the light passes through the moiré metasurfaces, it can be highly localized in the interval between the metasurfaces and show a strong chiral response. If the MO medium is located in it, the magneto‐optical effect can be enhanced. When *θ* = 90°, the guided mode disappears. This is also consistent with the above symmetry analysis.

Then, when an MF is applied to the MOMM, the complex amplitudes of spin states through MOMM are expressed as follows

(2) $$\left\{\begin{array}{c} \left|T_{R}=\left[{\left(A_{x}+A_{y}e^{i\varphi }\right)}^{2}t_{R}+{\left(A_{x}-A_{y}e^{i\varphi }\right)}^{2}t_{L}e^{-i2\theta }\right]/4\right.\\ \left|T_{L}=\left[{\left(A_{x}+A_{y}e^{i\varphi }\right)}^{2}t_{L}+{\left(A_{x}-A_{y}e^{i\varphi }\right)}^{2}t_{R}e^{-i2\theta }\right]/4\right. \end{array}\right.$$

where *A_x_* and *A_y_* are the linear polarized amplitudes along two main axes in the monolayer anisotropic metasurface, and *φ* is their phase difference. The details of the anisotropic optical responses for the single‐layer metasurface can be found in Section S3 of the Supporting Information.

$t_{R}=A_{R}e^{i\varphi_{R}}$is the complex amplitude of the *R* spin state through the InSb, and $t_{L}=A_{L}e^{i\varphi_{L}}$ is for the *L* spin state. The following discussion is a theoretical analysis of the MOMM, and more detailed derivations and discussions can be found in Section S4 of the Supporting Information.

When *θ* = 0°, ±90°, or 180°, put Equation (1) into Equation (2), we can get the same conclusion in the form of Equation (1), that is the nonreciprocal chirality with spin‐conjugate symmetry as shown in Figure 2b. When *θ* ≠ 0°, ±90°, or 180°, as illustrated in Figure 2c, this system has both MO chirality from InSb and structural chirality from the moiré metasurface, so the MOMM structure has broken both the space‐mirror and time‐reversal symmetry simultaneously, eliminating the spin‐conjugate symmetric transmission, which has a higher asymmetry degree than that of the two chiralities illustrated in Figure 2a,b, makes the four spin states to degenerate. The complete relations between the spin states through MOMM can be expressed as follows

(3) $$\begin{array}{cc} & \mathrm{Optical}\;\mathrm{Chirality}:T_{R+}\neq T_{L+},T_{R-}\neq T_{L-}\\ & \mathrm{Nonreciprocity}:T_{R+}\neq T_{R-},T_{L+}\neq T_{L-}\\ & \mathrm{Conjugate}\;\mathrm{Asymmetry}:T_{R+}\neq T_{L-},T_{R-}\neq T_{L+} \end{array}$$

When the following condition is satisfied at a contain twisted angle (called moiré angle *θ*_M_)

(4) $$-\frac{t_{L+}}{t_{R+}}{\left(\frac{A_{x}-A_{y}e^{i\varphi }}{A_{x}+A_{y}e^{i\varphi }}\right)}^{2}=e^{i2\theta_{M}}$$

the complex amplitudes of the four spin states are obtained as follows

(5) $$\left\{\begin{array}{c} \left|T_{R+}=0\right.\\ \left|T_{L+}=A_{x}A_{y}e^{i\varphi }\left(t_{L+}-t_{R+}e^{i2\theta_{M}}\right)\right.\\ \left|T_{R-}=\frac{1}{4}{\left(A_{x}+A_{y}e^{i\varphi }\right)}^{2}t_{L-}+\frac{1}{4}{\left(A_{x}-A_{y}e^{i\varphi }\right)}^{2}t_{R-}e^{i\left(-2\theta_{M}\right)}\right.\\ \left|T_{L-}=\frac{i}{2}{\left(A_{x}-A_{y}e^{i\varphi }\right)}^{2}t_{L-}\left(\sin2\theta_{M}\right)\right. \end{array}\right.$$

**Figure 3:**
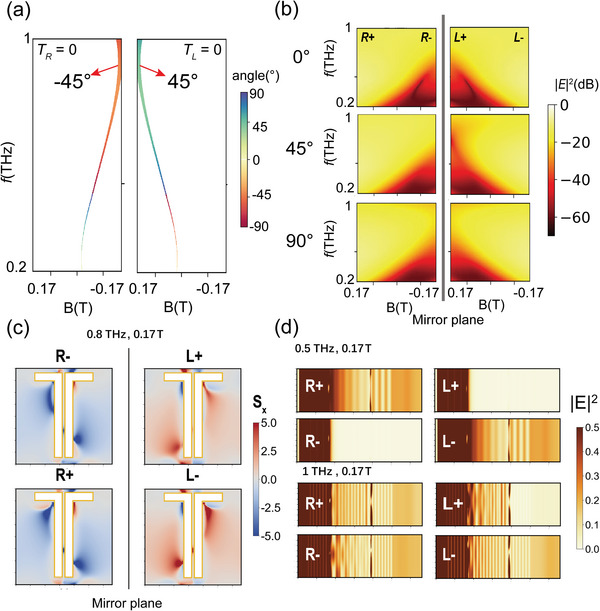
a) The calculated moiré angle *θ*_M_ for spin‐conjugate symmetry breaking under different MFs and THz frequencies. b) The calculated transmittance map of the MOMM with different MFs and THz frequencies for different twisted angles *θ* = 0°, 45°, and 90°. c) The simulation of the superchiral field for the incident spin *R*−, *L*+, *R*+, and *L*− in the MOMM with *θ* = 45°. The cross‐section is at the interface of the second metallic metasurface. d) The electric field propagation distributions through the MOMM with *θ* = 45° for the four incident spin states at 0.5 and 1 THz when *B* = 0.17 T.

In the condition of Equation (4), since *T_R_*_+_ = 0 and the other three spin states are not 0, the ideal chirality and nonreciprocal one‐way transmission with spin‐conjugate symmetry breaking can be obtained. For a fixed structure of anisotropic metasurface, we can rotate a certain angle *θ* to satisfy Equation (4) dependent on the corresponding MF, which is called the moiré angle *θ*_M_. The moiré angle *θ*_M_ under different frequencies and MFs can be obtained by Equation (4), and the calculated results are shown in **Figure** **3**a. In particular, when *θ*_M_ = 45° and Equation (4) is satisfied under *B* = 0.17 T around 0.8 THz, *T_L_*_−_ in Equation (5) can get the maximum value, so the strongest spin‐conjugate symmetry breaking *T_R_*_+_ ≠ *T_L_*_−_ can be obtained.

Since *A_x_*, *A_y_*, and *φ* can be optimally designed in the single layer anisotropic metasurface, and *t_R_* and *t_L_* of the InSb also can be broadly tuned by the different MFs. By reasonably matching the frequency positions of the chirality of the moiré metasurface and MO chirality of InSb, it is expected that the spin‐conjugate symmetry breaking condition can be approximately met in a broader frequency band, so the bandwidth of chiral or nonreciprocal one‐way transmission can be broadened or their intensity can be enhanced. We have theoretically calculated the transmittance map of MOMM with varying MF and frequency for the *R* and *L* states, as shown in Figure 3b. When *θ* = 0° and 90°, the transmittance of the *R* or *L* state itself is asymmetric to *B* = 0 T in the map, but their transmittance map is mirror‐antisymmetric (shown as mirror‐symmetry in the map): in the same direction of MF, the transmittances of the *R* or *L* state are different, but they are the same in the opposite direction of MF, which is just the spin‐conjugate symmetry. But when *θ* = 45°, this symmetry is broken: *T_R_*_+_ ≠ *T_L_*_−_ in the ≈0.6–1 THz range when the optimal *B* = ±0.17 T as shown in the middle subfigure of Figure 3b.

The superchiral field can quantitively describe the chiral enhancement in the device,^[^ ^50^ ^]^ and the chirality enhancement index *S*_*χ*_ of the superchiral field in the *x‐y* cutting plane can be calculated as follows

(6) $$S_{\chi }=-\frac{\mathrm{cIm}\left[{\tilde{E}}^{*}\tilde{B}\right]}{{\left|E_{\mathrm{CPL}}\right|}^{2}}$$

where *E* and *B* are the local electric and magnetic fields, E* denotes the complex conjugate of the electric field, and *E*_CPL_ is the electric field of a plane circularly polarized wave. The details of the superchiral field can be found in Section S5 of the Supporting Information. We simulate the chirality enhancement index *S*_*χ*_ of the superchiral field distribution of MOMM with *θ* = 45° at the interface of the second metallic metasurface, as shown in Figure 3c. The *L*+ and *R*− show asymmetry in the superchiral field, which indicates that the superchiral field originating from the moiréstructure interacts with InSb, leading to different chiral responses and removing degeneracy between *L*+ and *R*−. For *L*− and *R*+, the superchiral field exhibits mirror‐antisymmetry, which means these two spin states are still degenerate at *f* = 0.8 THz, *B* = ±0.17 T. We also simulate the electric field propagation distributions of MOMM as shown in Figure 3d. At the cyclotron resonance frequency of 0.5 THz, *R*− and *L*+ are forbidden, but both *R*+ and *L*− can transmit. Far from the cyclotron resonance frequency of 1 THz, *L*+ is forbidden, but *R*− can transmit, and both *R*+ and *L*− can transmit, showing spin‐conjugate symmetry breaking.

### 2.2 Experimental Results

**Figure 4:**
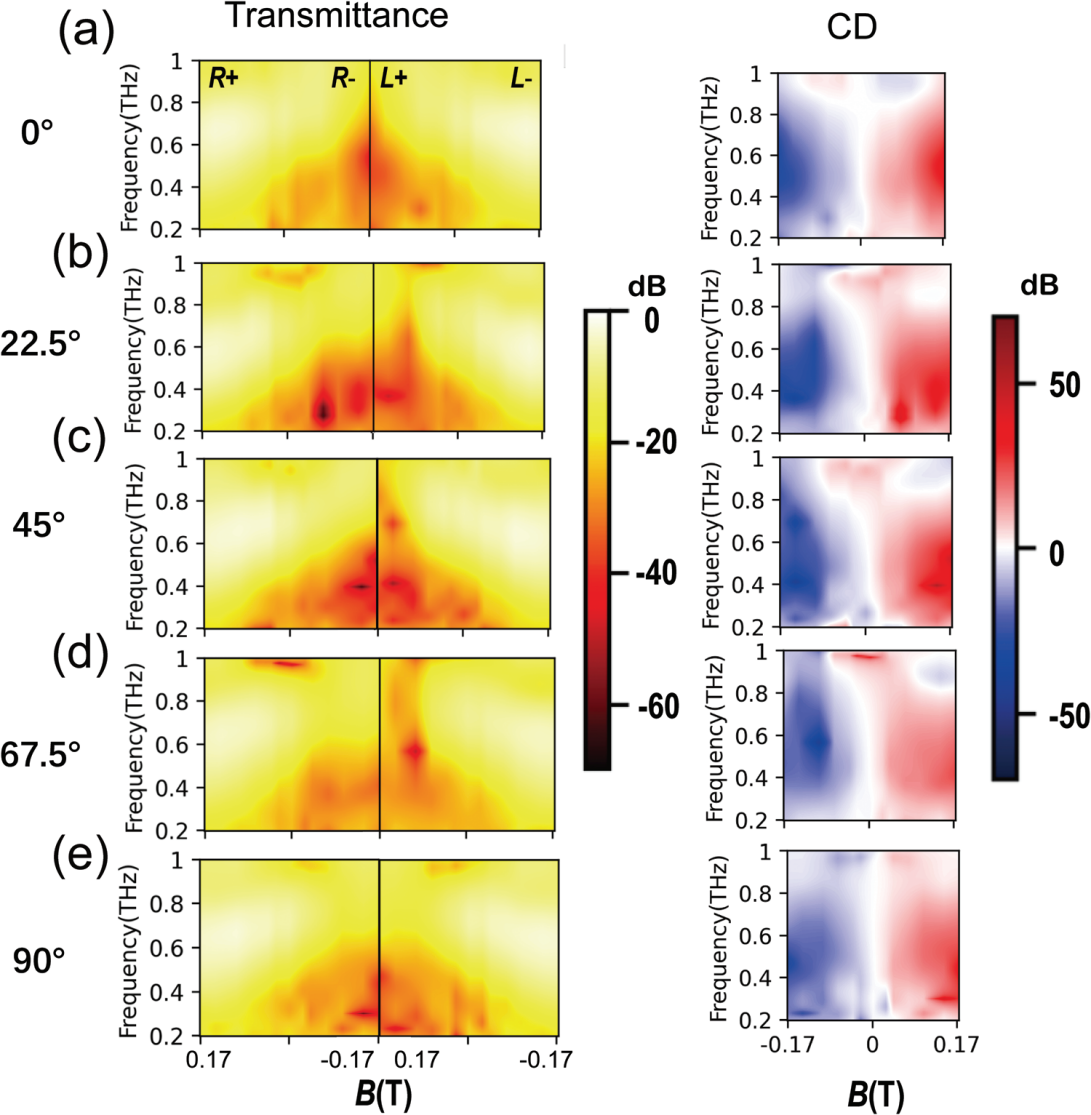
The experimental a) transmittance map and b) the corresponding CD map of the MOMM for different twisted angles *θ* = 0°, 22.5°, 45°, 67.5°, and 90° with varying MFs and THz frequencies measured by the THz‐TDMPS system.

We apply the terahertz time‐domain magneto polarization spectroscopy (THz‐TDMPS) to measure the MOMM, the details of the experimental system and methods can be found in the Experimental Section; and Section S1 of the Supporting Information. We obtain the experimental transmittance map in **Figure** **4**a, and the experimental results for *θ* = 0°, 45°, and 90° are consistent with the theoretical calculation results of the transmission matrix in Figure 3b. Meanwhile, we can also notice that when *θ* = 22.5° and 67.5°, their map is mirror‐asymmetrical like 45°. Their conjugate symmetry breaking still has a wide bandwidth from 0.6 to 1 THz, but the optimal biased MF drops to 0.1 THz since the moiré angle changes.

We define *CD*(*B*, *f*) = *T_R_* (*B*, *f*) − *T_L_* (*B*, *f*) to characterize the chiral response of the device and obtain the corresponding CD map of the MOMM in Figure 4b. When *θ* = 0° and 90°, the CD map is mirror‐antisymmetric to *B* = 0, which means the chiral spin state is reverse but in equal strength under the opposite direction of MF. The maximum CD is 28 dB. When *θ* = 22.5°, 45°, and 67.5°, the intensity of CD becomes asymmetric to *B* = 0. Take 45° as an example, in the region of *B* > 0, the bandwidth of the red zone is narrowed, but its intensity increases to 50 dB. In the *B* < 0 region, the blue region widens, and its 10 dB bandwidth increases to more than 700 GHz, so the CD value is asymmetric to *B* = 0. The introduction of the moiré metasurface structure manipulates the MO effect of InSb, which enhances the chiral response in one MF direction (or propagation direction) and expands the chirality bandwidth in the other opposite direction. Meanwhile, reducing the degeneracy of spin states enriches the transmission states of the system.

**Figure 5:**
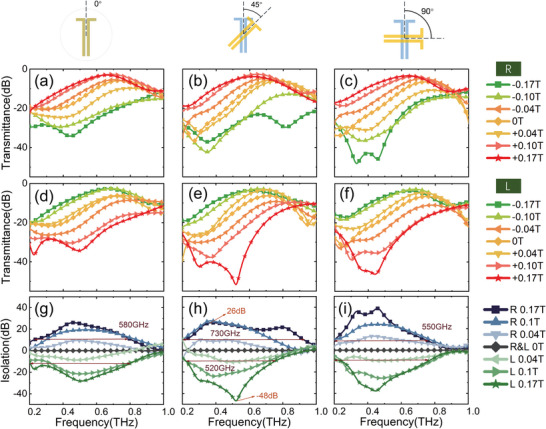
The experimental transmission spectra of different MOMM for different spin state under several MFs: *T*_R_ with a) *θ* = 0°, b) 45°, and c) 90°; *T*_L_ with d) *θ* = 0°, e) 45°, and f) 90°. The corresponding isolation degree for *R* and *L* spin state of MOMM with d) *θ* = g) 0°, h) 45°, and i) 90°.

To quantitatively analyze the nonreciprocity and spin‐conjugate symmetry, we select several MFs to plot the transmission spectra for *R* and *L* states at different twisted angles in **Figure** **5**. As shown in Figure 5a,d and e,f, when *θ* = 0° and 90°, nonreciprocal one‐way transmission is exhibited for one of the spin states. For example, at 0.65 THz in Figure 5a,d, the *R* state transmits through the device with *T_R_*_+_ = −2.6 dB but forbids with *T_R_*_−_ = −25 dB, and the *L* state transmits through the device with *T_L_*_−_ = −2.6 dB but forbids with *T_L+_* = −25 dB. But it is spin‐conjugate symmetric to the MF between *R* and *L* because *T_R_*_+_ = *T_L_*_−_ and *T_R_*_−_ = *T_L+_*. When *θ* = 45° in Figure 5b,e, *T_R_*_−_ and *T_L_*_+_ become different at *B* = 0.17 T. for example at 0.52 THz, *T_R_*_−_ = −25 dB but *T_L+_* = −51 dB. The corresponding simulated transmission spectra can be found in Section S6 of the Supporting Information, which confirms the above experimental results.

Next, we define the isolation degree (expressed in dB) to characterize nonreciprocity and one‐way transmission of the device as follows for the *R* and *L* state, respectively^[^ ^51^ ^]^

(7) $$\begin{array}{cc} & I{\mathrm{so}}_{R}\left(\mathrm{dB}\right)=10\log\left[T_{R+}\left(\%\right)/T_{R-}\left(\%\right)\right]=T_{R+}\left(\mathrm{dB}\right)-T_{R-}\left(\mathrm{dB}\right)\\ & I{\mathrm{so}}_{L}\left(\mathrm{dB}\right)=10\log\left[T_{L+}\left(\%\right)/T_{L-}\left(\%\right)\right]=T_{L+}\left(\mathrm{dB}\right)-T_{L-}\left(\mathrm{dB}\right) \end{array}$$

where *I*so > 0 indicates that the backward transmission is isolated, and *I*so < 0 means that forward transmission is forbidden. The calculated results are as shown in Figure 5g–i. It can be seen that when *θ* = 0° in Figure 5g, these curves are mirror‐antisymmetric to the MFs on the isolation spectra, which means that the *R* state can pass through but the *L* state is isolated under the positive MF, while the *L* state passes through but the *R* state is forbidden under the reverse MF. The isolation degree can reach 30 dB at 0.5 THz, and the isolation band with a 10 dB isolation degree is in the range of 0.25–0.8 THz (i.e., 550 GHz bandwidth). The case of *θ* = 90° in Figure 5i is similar, only the isolation is increased to 40 dB. When *θ* = 45° in Figure 5h, the isolation spectra of *R* and *L* states are asymmetric to *B* = 0, indicating the spin‐conjugate symmetry breaking. The isolation bandwidth of the *R* state increases up to 730 GHz (in the range of 0.2–0.93 THz with 129.2% relative bandwidth), meanwhile, the isolation degree of the *L* state increase to 48 dB although its bandwidth is slightly reduced. In particular, in the frequency range of 0.75–1 THz, only the *R* state can transmit unidirectional, while both the forward and reverse *L* states pass through the MOMM. Therefore, the breaking of spin‐conjugate symmetry originating from the moiré metasurface structure leads to an enhanced isolation degree of nonreciprocal one‐way transmission in one spin state and an extended isolation bandwidth in the other spin state. In addition, although the isolation effect is worked for circular polarizations, for the linear polarization commonly used in the THz system, the isolation effect can be also achieved by adding the additional THz polarizers and wave‐plates.^[^ ^51^ ^]^

**Figure 6:**
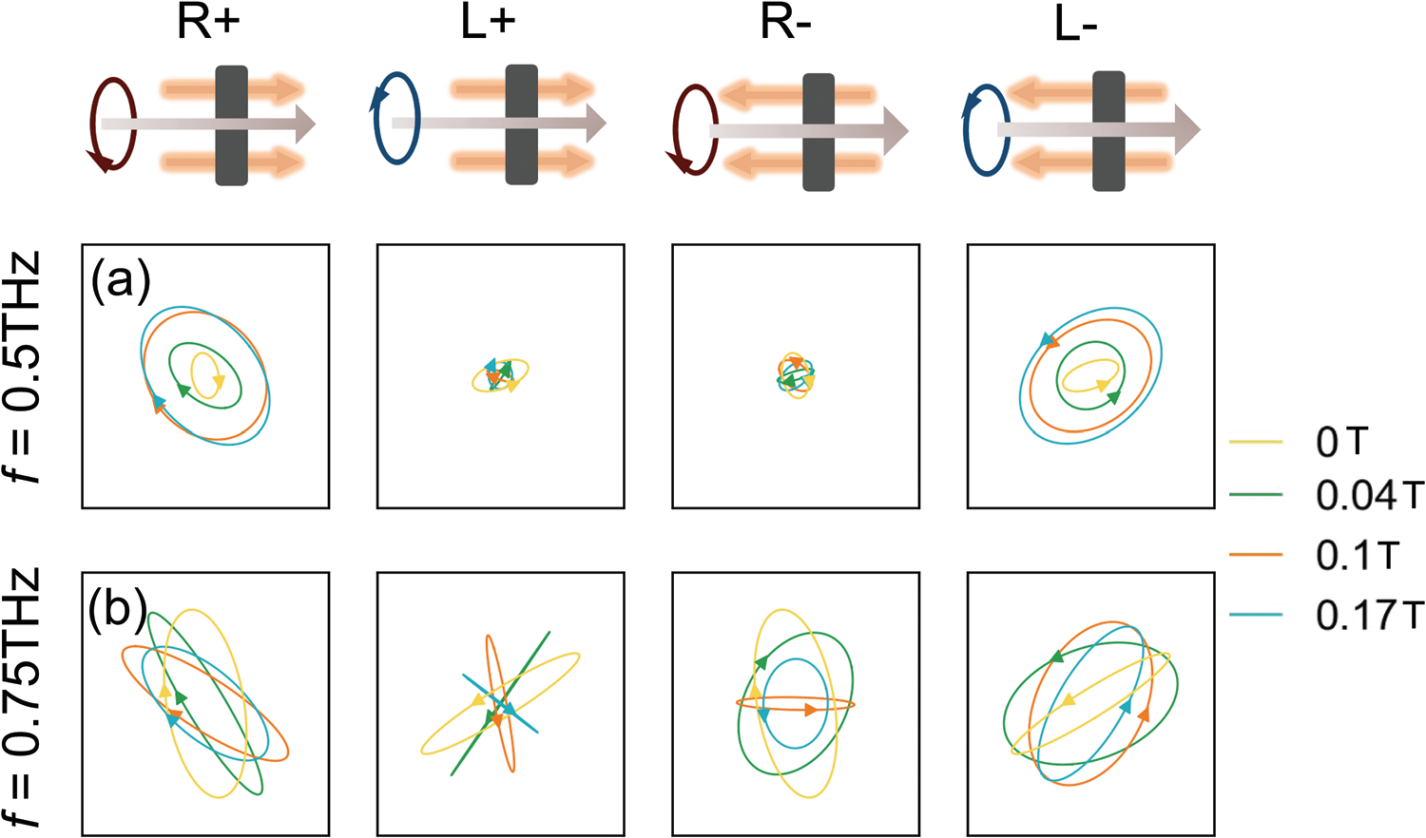
The polarization ellipse of the four incident spin states *R*+, *L*+, *R*−, and *L*− for MOMM with *θ* = 45° under the different MFs at a) 0.5 THz and b) 0.75 THz.

To further demonstrate the nonreciprocal chirality effect described above, we plot the polarization ellipse under the different MFs for the MOMM with *θ* = 45° in **Figure** **6**. In the cyclotron resonance band, e.g., at *f* = 0.5 THz in Figure 6a, the intensity of the polarization ellipsoid for both *L*+ and *R*− is very small, which indicate these two spin states are forbidden to pass through the MOMM. The intensity of *L*− and *R*+ gradually increases with the MF, and the output electric field is the same circularly polarized state as the incident spin state. However, when the frequency is far from the cyclotron resonance frequency *ω*_c_, e.g., at *f* = 0.75 THz in Figure 6b, the output polarization responses of the four input spin states are all not 0 but quite different, which shows the spin‐conjugate symmetry breaking. For example, as the MF increases, the output polarization state of the *R*− tends to be circularly polarized, but for *L*+, the outputting light tends to be linearly polarized and rotating with the increase of the MF.

## 3 Conclusion

In summary, we develop a THz MO moiré metasurface to realize the active chirality and one‐way spin transmission with the spin‐conjugate symmetry breaking, and there are some important points as follows: 1) Like the moiré superlattice in the electronic regime, the spatial inversion or mirror symmetry breaking introduces the chiral response for the spin photons, and then the time‐reversal symmetry breaking achieves with the gyrotropic semiconductor under the MFs. Combining these two symmetries breaking, a moiré angle is demonstrated in which case the nonreciprocity and chirality with spin‐conjugate asymmetric transmission are obtained in this device. This MO photonic moiré structure is of great value for the analogy of the electron spin behavior with the space‐time symmetry‐breaking effect in the nanoscale moiré superlattice system. 2) Based on this mechanism, the experiment and simulation confirm that the THz chirality between the two conjugate spin states can be independently manipulated under the positive and negative MF or propagation directions, and such reducing degeneracy of spin states provides a new way for spin multiplexing and complex coding. 3) Meanwhile, the two spin states also realize the nonreciprocal one‐way transmission, and their isolation spectra are also spin‐conjugate asymmetric: one is enhanced up to 48 dB, and the other's bandwidth is widened to over 0.73 THz both with a low insertion loss of 3 dB. This provides a new way to improve the isolation degree and bandwidth of THz isolators. Therefore, this MOMM device and its mechanism in time‐space asymmetric transmission have promising potential in fundamental physics and THz application systems.

## 4 Experimental Section

The metallic metasurfaces are fabricated on SiO_2_ substrate by the metal evaporation, UV lithography, and lift‐off process. <111> *n*‐InSb single crystals are prepared by liquid phase epitaxy with the intrinsic carrier density of *N* = 2 × 10^14^ cm^−3^. The front and back surfaces of InSb are electrostatically adsorbed on the metallic metasurface, and there is a twisted angle *θ* between two layers of metallic metasurface patterns, which forms the MOMM with a moiré pattern. Finally, this compound structure is encapsulated with UV glue.

The experiment was conducted by the terahertz time‐domain magneto polarization spectroscopy (THz‐TDMPS) system. The THz signal is generated by the photoconductive antenna (PCA) with an 800 nm femtosecond laser pumping. In the receiver port, a (110) ZnTe crystal is used for the electro‐optical detection probed by the *y*‐direction linear polarized femtosecond laser. Therefore, the (001) axis of the ZnTe is rotated along the *x*‐axis to get the best efficiency. Then, a couple of additional polarizers are placed both in front of and behind the sample to adjust the polarization states of both the incident and received signals. Both the front side and the back side polarizer can be rotated to an arbitrary angle to control the polarization of light. The details of the Experimental Section can be found in Section S1 of the Supporting Information.

Numerical simulations were performed by using the finite time domain difference (FDTD) method by commercial software, Lumerical FDTD Solutions 2020 R2, including photonic band structures as shown in Figure 2e, field distributions in Figure 3c,d of the main text, and transmission spectra in Figure S4 of the Supporting Information. For the photonic band structure calculation, the Bloch boundary conditions are applied for the *x* and *y* directions and the PML boundary condition is used for the *z* direction. All possible modes of the system are excited by using multiple randomly placed broadband dipoles. The wave vector *k* is specified by the Bloch boundary conditions, which means one simulation per *k*‐vector is required. At frequencies where a mode (i.e., the photonic band) exists, the fields will propagate indefinitely. At all other frequencies, the fields will quickly disappear due to destructive interference. Then, a set of dipole sources are applied in the unit cell with time monitors to detect the response. Finally, the band diagram can be get by the proposed analysis group. For the field distribution and transmission spectra simulations, the periodic boundary conditions are applied for the *x* and *y* directions and the PML boundary condition for the *z* direction. The plane wave sources incident into the structure and the signals can be detected with the 2D DFT detector. Here, the first metallic metasurface with 0 rotational angles is set as depicted in Figure 1c, and the material is PEC. The second metallic metasurface with rotational angle *θ* is set as the periodical structure with the S‐parameter equivalence method, making sure that *t*_2_(*x*, *y*) = *t*_1_(*x*sin*θ*, *y*cos*θ*), where *t*_1_, *t*_2_ denotes the transmission functions for the first and second metasurfaces. InSb as the Sampled 3D data with the calculated dielectric parameters of the dielectric tensor, and a Matrix transform is applied to make the material gyrotropic was set. The SiO_2_ substrate is set as a loss‐free material with a refractive index of 1.9, which is consistent with its experimental data in 0.2–1 THz.

## Conflict of Interest

The authors declare no conflict of interest.

## Supporting information

Supporting Information

## Data Availability

The data that support the findings of this study are available from the corresponding author upon reasonable request.

## References

[1] K. W. Bentley , Y. G. Nam , J. M. Murphy , C. Wolf , J. Am. Chem. Soc. 2013, 135, 18052. 24261969 10.1021/ja410428b

[2] A. Ben‐Moshe , B. M. Maoz , A. O. Govorov , G. Markovich , Chem. Soc. Rev. 2013, 42, 7028. 23788027 10.1039/c3cs60139k

[3] A. O. Govorov , Z. Fan , P. Hernandez , J. M. Slocik , R. R. Naik , Nano Lett. 2010, 10, 1374. 20184381 10.1021/nl100010v

[4] D. G. Grier , Nature 2003, 424, 810. 12917694 10.1038/nature01935

[5] J. F. Lawler , J. G. Lunney , J. M. D. Coey , Appl. Phys. Lett. 1994, 65, 3017.

[6] J. A. Gaj , R. R. Gata̧zka , M. Nawrocki , Solid State Commun. 1993, 88, 923.

[7] M. Inoue , MRS Online Proc. Libr. 2004, 853, 131.

[8] J. P. van der Ziel , P. S. Pershan , L. D. Malmstrom , Phys. Rev. Lett. 1965, 15, 190.

[9] W.‐K. Tse , A. H. MacDonald , Phys. Rev. Lett. 2010, 105, 057401. 20867952 10.1103/PhysRevLett.105.057401

[10] B. Briat , C. Djerassi , Nature 1968, 217, 918.

[11] F. D. M. Haldane , S. Raghu , Phys. Rev. Lett. 2008, 100, 013904. 18232766 10.1103/PhysRevLett.100.013904

[12] D. Floess , J. Y. Chin , A. Kawatani , D. Dregely , H.‐U. Habermeier , T. Weiss , H. Giessen , Light: Sci. Appl. 2015, 4, e284,

[13] V. V. Temnov , G. Armelles , U. Woggon , D. Guzatov , A. Cebollada , A. Garcia‐Martin , J.‐M. Garcia‐Martin , T. Thomay , A. Leitenstorfer , R. Bratschitsch , Nat. Photonics 2010, 4, 107.

[14] Z. Wang , Y. Chong , J. D. Joannopoulos , M. Soljačić , Nature 2009, 461, 772. 19812669 10.1038/nature08293

[15] M. Shalaby , M. Peccianti , Y. Ozturk , R. Morandotti , Nat. Commun. 2013, 4, 1558. 23463001 10.1038/ncomms2572 PMC3615378

[16] J. Qin , L. Deng , T. Kang , L. Nie , H. Feng , H. Wang , R. Yang , X. Liang , T. Tang , J. Shen , C. Li , H. Wang , Y. Luo , G. Armelles , L. Bi , ACS Nano 2020, 14, 2808. 32074454 10.1021/acsnano.9b05062

[17] I. Zubritskaya , N. Maccaferri , X. I. Ezeiza , P. Vavassori , A. Dmitriev , Nano Lett. 2018, 18, 302. 29240446 10.1021/acs.nanolett.7b04139

[18] E. Keshock , P. Peng , J. Zhou , D. Talbayev , Opt. Express 2020, 28, 38280. 33379643 10.1364/OE.411581

[19] Z. Li , Y. Cheng , H. Luo , F. Chen , X. Li , J. Alloys Compd. 2022, 925, 166617.

[20] B. He , J. Liu , Y. Cheng , F. Chen , H. Luo , X. Li , Phys. E 2022, 144, 115373.

[21] Y. Cheng , Z. Li , Z. Cheng , Opt. Mater. 2021, 117, 111129.

[22] S. Lin , S. Silva , J. Zhou , D. Talbayev , Adv. Opt. Mater. 2018, 6, 1800572.

[23] D. Wang , B. Yang , W. Gao , H. Jia , Q. Yang , X. Chen , M. Wei , C. Liu , M. Navarro‐Cía , J. Han , W. Zhang , S. Zhang , Nat. Phys. 2019, 15, 1150.

[24] S. M. Hanham , A. I. Fernández‐Domínguez , J. H. Teng , S. S. Ang , K. P. Lim , S. F. Yoon , C. Y. Ngo , N. Klein , J. B. Pendry , S. A. Maier , Adv. Mater. 2012, 24, OP226. 22807039 10.1002/adma.201202003

[25] T. Li , F. Fan , Y. Ji , Z. Tan , Q. Mu , S. Chang , Opt. Lett. 2020, 45, 1.

[26] Q. Mu , F. Fan , S. Chen , S. Xu , C. Xiong , X. Zhang , X. Wang , S. Chang , Photon. Res. 2019, 7, 325.

[27] Y. Cao , V. Fatemi , A. Demir , S. Fang , S. L. Tomarken , J. Y. Luo , J. D. Sanchez‐Yamagishi , K. Watanabe , T. Taniguchi , E. Kaxiras , R. C. Ashoori , P. Jarillo‐Herrero , Nature 2018, 556, 80. 29512654 10.1038/nature26154

[28] Y. Cao , V. Fatemi , S. Fang , K. Watanabe , T. Taniguchi , E. Kaxiras , P. Jarillo‐Herrero , Nature 2018, 556, 43. 29512651 10.1038/nature26160

[29] M. Yankowitz , S. Chen , H. Polshyn , Y. Zhang , K. Watanabe , T. Taniguchi , D. Graf , A. F. Young , C. R. Dean , Science 2019, 363, 1059. 30679385 10.1126/science.aav1910

[30] J. Ahn , S. Park , B.‐J. Yang , Phys. Rev. X 2019, 9, 021013.

[31] K. P. Nuckolls , M. Oh , D. Wong , B. Lian , K. Watanabe , T. Taniguchi , B. A. Bernevig , A. Yazdani , Nature 2020, 588, 610. 33318688 10.1038/s41586-020-3028-8

[32] M. J. Park , Y. Kim , G. Y. Cho , S. Lee , Phys. Rev. Lett. 2019, 123, 216803. 31809156 10.1103/PhysRevLett.123.216803

[33] K. L. Seyler , P. Rivera , H. Yu , N. P. Wilson , E. L. Ray , D. G. Mandrus , J. Yan , W. Yao , X. Xu , Nature 2019, 567, 66. 30804526 10.1038/s41586-019-0957-1

[34] E. M. Alexeev , D. A. Ruiz‐Tijerina , M. Danovich , M. J. Hamer , D. J. Terry , P. K. Nayak , S. Ahn , S. Pak , J. Lee , J. I. Sohn , M. R. Molas , M. Koperski , K. Watanabe , T. Taniguchi , K. S. Novoselov , R. V. Gorbachev , H. S. Shin , V. I. Fal'ko , A. I. Tartakovskii , Nature 2019, 567, 81. 30842637 10.1038/s41586-019-0986-9

[35] T. I. Andersen , G. Scuri , A. Sushko , K. De Greve , J. Sung , Y. Zhou , D. S. Wild , R. J. Gelly , H. Heo , D. Bérubé , A. Y. Joe , L. A. Jauregui , K. Watanabe , T. Taniguchi , P. Kim , H. Park , M. D. Lukin , Nat. Mater. 2021, 20, 480. 33398121 10.1038/s41563-020-00873-5

[36] T. Huang , X. Tu , C. Shen , B. Zheng , J. Wang , H. Wang , K. Khaliji , S. H. Park , Z. Liu , T. Yang , Z. Zhang , L. Shao , X. Li , T. Low , Y. Shi , X. Wang , Nature 2022, 605, 63. 35508778 10.1038/s41586-022-04520-8

[37] J. D. Joannopoulos , S. G. Johnson , J. N. Winn , R. D. Meade , Photonic Crystals: Molding the Flow of Light, Princeton University Press, Princeton, NJ 2008.

[38] M. Renuka , X. Lin , Z. Wang , L. Shen , B. Zheng , H. Wang , H. Chen , Opt. Lett. 2018, 43, 5737. 30499981 10.1364/OL.43.005737

[39] G. Hu , A. Krasnok , Y. Mazor , C.‐W. Qiu , A. Alù , Nano Lett. 2020, 20, 3217. 32298129 10.1021/acs.nanolett.9b05319

[40] T. Jiang , H. Liu , D. Huang , S. Zhang , Y. Li , X. Gong , Y.‐R. Shen , W.‐T. Liu , S. Wu , Nat. Nanotechnol. 2014, 9, 825. 25173830 10.1038/nnano.2014.176

[41] M. Chen , X. Lin , T. H. Dinh , Z. Zheng , J. Shen , Q. Ma , H. Chen , P. Jarillo‐Herrero , S. Dai , Nat. Mater. 2020, 19, 1307. 32661384 10.1038/s41563-020-0732-6

[42] Z. Zheng , F. Sun , W. Huang , J. Jiang , R. Zhan , Y. Ke , H. Chen , S. Deng , Nano Lett. 2020, 20, 5301. 32574060 10.1021/acs.nanolett.0c01627

[43] J. Duan , N. Capote‐Robayna , J. Taboada‐Gutiérrez , G. Álvarez‐Pérez , I. Prieto , J. Martín‐Sánchez , A. Y. Nikitin , P. Alonso‐González , Nano Lett. 2020, 20, 5323. 32530634 10.1021/acs.nanolett.0c01673

[44] T. Stauber , T. Low , G. Gómez‐Santos , Phys. Rev. Lett. 2018, 120, 046801. 29437442 10.1103/PhysRevLett.120.046801

[45] X. Mu , M. Sun , Mater. Today Phys. 2020, 14, 100222.

[46] N. V. Tepliakov , A. V. Orlov , E. V. Kundelev , I. D. Rukhlenko , J. Phys. Chem. C 2020, 124, 22704.

[47] Q. Fu , P. Wang , C. Huang , Y. V. Kartashov , L. Torner , V. V. Konotop , F. Ye , Nat. Photonics 2020, 14, 663.

[48] P. Wang , Y. Zheng , X. Chen , C. Huang , Y. V. Kartashov , L. Torner , V. V. Konotop , F. Ye , Nature 2020, 577, 42. 31853062 10.1038/s41586-019-1851-6

[49] Z. Tan , F. Fan , D. Zhao , Y. Ji , J. Cheng , S. Chang , Adv. Opt. Mater. 2021, 9, 2002216.

[50] Y. Tang , A. E. Cohen , Science 2011, 332, 333. 21493854 10.1126/science.1202817

[51] M. Tamagnone , C. Moldovan , J.‐M. Poumirol , A. B. Kuzmenko , A. M. Ionescu , J. R. Mosig , J. Perruisseau‐Carrier , Nat. Commun. 2016, 7, 11216. 27048760 10.1038/ncomms11216 PMC4823866

