# Supplementary material for: Terahertz Spin‐Conjugate Symmetry Breaking for Nonreciprocal Chirality and One‐Way Transmission Based on Magneto‐Optical Moiré Metasurface
Source: Adv Sci (Weinh). 2022 Nov 14;10(4):2204916. doi: 10.1002/advs.202204916 (PMC9896033; doi:10.1002/advs.202204916)
Supplement: Supplementary file 1 — Supporting Information [file ADVS-10-2204916-s001.pdf]

Supporting Information

Terahertz spin-conjugate symmetry breaking for nonreciprocal chirality and one-way transmission based on magneto-optical moiré metasurface

Zhiyu Tan, Fei Fan, Shengnan Guan, Hao Wang, Dan Zhao, Yunyun Ji, Shengjiang Chang **

Z. Tan, F. Fan, S. Guan, H. Wang
Institute of Modern Optics, Nankai University, Tianjin Key Laboratory of Micro-scale Optical Information Science and Technology, Tianjin 300350, China
E-mail: (fanfei@nankai.edu.cn)

D. Zhao, Y. Ji, S. Chang
Tianjin Key Laboratory of Optoelectronic Sensor and Sensing Network Technology, Tianjin 300350, China
E-mail: (sjchang@nankai.edu.cn)

S1. Experiment Setup and Methods
S2. THz magneto-optical properties of bulk InSb
S3. Anisotropic optical response of monolayer metasurface
S4. The theoretical calculation for MOMM and conjugate symmetry breaking
S5. Superchiral field
S6. Simulation results of the MOMM
References

S1. Experiment Setup and Methods

In this work, we use the terahertz time-domain magneto polarization spectroscopy (THz-TDMPS) system for the experiment. The photograph of the experiment system is shown in Fig. S1(a). Here, the THz signal is generated by the photoconductive antenna with an 800nm femtosecond laser pumping. Then, a couple of additional polarizers are placed both in front of and behind the sample to adjust the polarization states of both the incident and received signals as shown in Fig. S1(b). And in the receiver port, a (110) ZnTe crystal is used for the electro-optical detection probed by the y-direction linear polarized (LP) femtosecond laser. Therefore, the (001) axis of the ZnTe is rotated along the x-axis to get the best efficiency.[1] Both the front side and the back side polarizer can be rotated to an arbitrary angle to control the polarization of light. If the first polarizer is rotated to a couple of orthogonal angles, and as same as the second, we can get four LP components (*e.g.* from x to x, x to y, y to x, y to y). After Fourier transforms, we can get the transmission and phase spectra of arbitrary LP state to arbitrary LP component.

When the polarizers are rotated to ±45°, we can obtain different linearly polarized time-domain pulse signals for the MOMM structure under different magnetic fields (MFs). After Fourier transforms, we can get the relative electric field for the sample in the frequency domain $\tilde{E}_{ab}(f) = A_{ab}(f)e^{i\theta_{ab}} / |A_r(f)|$, where the subscript a, b = x or y denotes the incident and output polarization state, respectively, and r denotes the reference data (Dewar flask without any object inside). If the orthogonal LP components are transformed into the orthogonal circular polarization (CP) basis vectors, we can get the electric field of the two spin components as follows:

$$\begin{aligned} \tilde{E}_R &= \left[\tilde{E}_{xx} + \tilde{E}_{yy} - i(\tilde{E}_{yx} - \tilde{E}_{xy}) \right] / 2 \\ \tilde{E}_L &= \left[\tilde{E}_{xx} + \tilde{E}_{yy} + i(\tilde{E}_{yx} - \tilde{E}_{xy}) \right] / 2 \end{aligned} \quad \text{(S1)}$$

To fully represent the arbitrary polarization state of the output light, the terminal trajectory equation of electric vector E, also called the polarization ellipse. Here, if the incident light is in CP state, we detect the orthogonal LP components E_x and E_y, so the polarization ellipse can be obtained as follows:

$$|E_x|^2 + |E_y|^2 - 2|E_x||E_y|\cos\Delta\delta = \sin^2\Delta\delta \quad \text{(S2)}$$

where $\Delta\delta = \varphi_y - \varphi_x$ is the phase difference between these orthogonal states. According to the Eq. S2, we can get the results of Figure 6 in the main text from the experimental data.

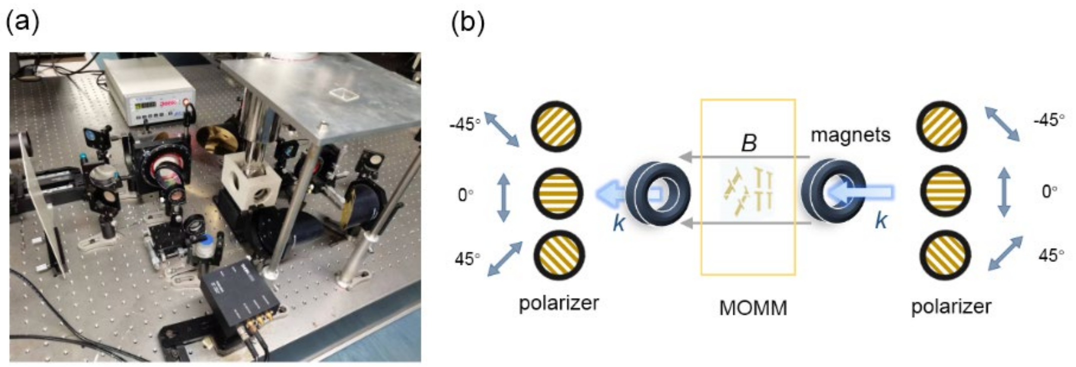

Fig. S1. (a) Photograph of the THz-TDMPS system. (b) Sketch map of the polarizers and the detection of different polarizations.

S2. THz magneto-optical properties of bulk InSb

The InSb is a gyroelectric semiconductor when a biased magnetic field is applied. In our work, the biased MF is along the z-axis, Maxwell's wave equation can be expressed in a nonreciprocal tensor as follows:

$$-\beta^2\begin{bmatrix}E_x\\E_y\\E_z\end{bmatrix}+\begin{bmatrix}0\\0\\\beta^2E_z\end{bmatrix}+\omega^2\mu_0\varepsilon_0\begin{bmatrix}\varepsilon_1 & -i\varepsilon_2 & 0\\ i\varepsilon_2 & \varepsilon_1 & 0\\ 0 & 0 & \varepsilon_3\end{bmatrix}\begin{bmatrix}E_x\\E_y\\E_z\end{bmatrix}=0, \tag{S3}$$

where the ε_1 and ε_2 can be written as:[2]

$$\begin{aligned}\varepsilon_1&=\varepsilon_\infty-\frac{\omega_p^2(\omega+\gamma i)}{\omega[(\omega+\gamma i)^2-\omega_c^2]}\\ \varepsilon_2&=-\frac{\omega_p^2\omega_c}{\omega[(\omega+\gamma i)^2-\omega_c^2]}\end{aligned} \tag{S4}$$

where ω_c is the cyclotron frequency that is proportional to the magnetic field by $\omega_c = eB/m^*$, B is the magnetic flux density, e is the electron charge, m^* is the effective mass of the carrier. For the InSb, $m^* = 0.014m_e$, and m_e is the mass of the electron. $\varepsilon_\infty = 15.68$ is the high-frequency limit permittivity; ω is the circular frequency of the incident THz wave; ω_p is plasma frequency written as $\omega_p = (Ne^2/m^*\mu)^{1/2}$, γ is the collision frequency of carriers, $\gamma = 4*e/(\mu m^*)$, and μ is the carrier mobility, which can be modeled as $\mu = 7.7\times10^4\ (T/300)^{-1.66}$ $cm^2 \cdot V^{-1} \cdot s^{-1}$.[2-4] All the theories and experiments for InSb are performed at $T = 80K$ in this work, where the intrinsic carrier concentration of InSb $N = 2\times10^{14}\,\mathrm{cm}^{-3}$.[5-7]

Two eigen photonic spin states, that is left-handed (L) and right-handed (R) spin states, can be solved from Eq. S3:

$$\begin{aligned}\beta_L&=\omega\sqrt{\mu_0\varepsilon_L},\varepsilon_L=(\varepsilon_1-\varepsilon_2),E_y=-iE_x,\\ \beta_R&=\omega\sqrt{\mu_0\varepsilon_R},\varepsilon_R=(\varepsilon_1+\varepsilon_2),E_y=iE_x,\end{aligned} \tag{S5}$$

Thus, the Jones matrix for the InSb under the CP basis can be written as:

$$T_{InSb}=\begin{bmatrix}t_R & t_{RL}\\ t_{LR} & t_L\end{bmatrix}=\begin{bmatrix}A_Re^{i\varphi_R} & 0\\ 0 & A_Le^{i\varphi_L}\end{bmatrix} \tag{S6}$$

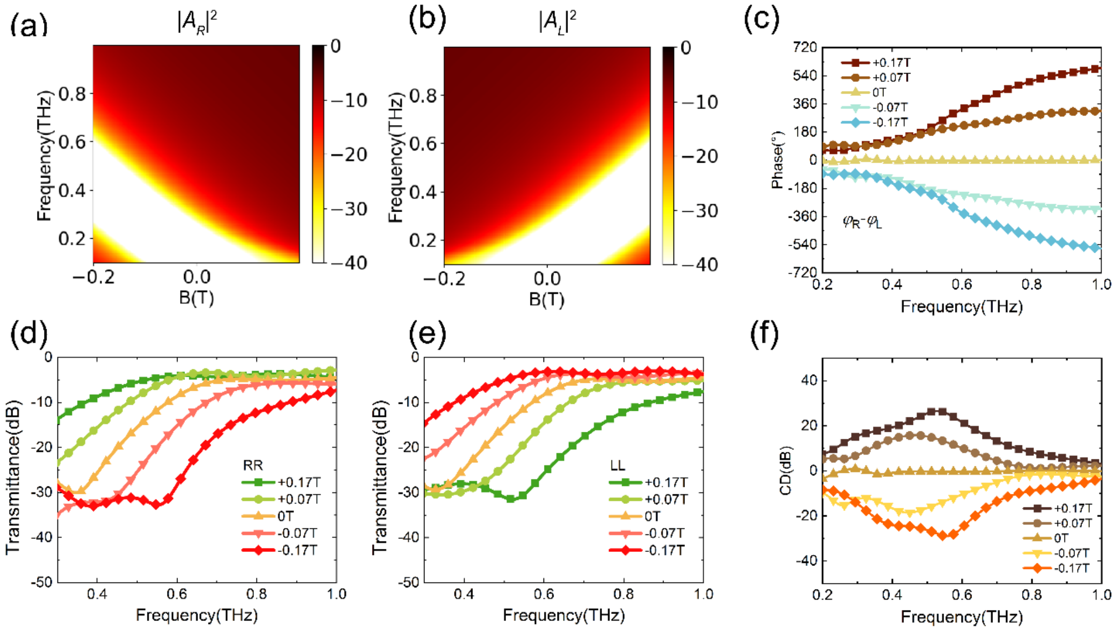

Fig. S2. The calculated transmittance map of the InSb under different MFs for (a) L and (b) R spin states. (c) The experimental phase difference between the R and L state under the different MFs. The experimental transmission spectra of bulk InSb for the (d) R and (e) L states. (f) The experimental CD spectra under the different MFs.

There is no conversion between conjugated spin states, so the spin-flip states both t_{RL} and t_{LR}=0, and both L and R state only refer to spin-locked states. Then we can theoretically calculate the transmittance and phase of the L or R state through InSb according to the Fresnel formula:

$$\begin{aligned} |A_L|^2 &= \frac{4\,\mathrm{Re}(\sqrt{\varepsilon_L})}{[\mathrm{Re}(\sqrt{\varepsilon_L})+1]^2}\exp\left[-\frac{2\mathrm{Im}(\sqrt{\varepsilon_L})\omega d}{c}\right], \varphi_L = \beta_L d \\ |A_R|^2 &= \frac{4\,\mathrm{Re}(\sqrt{\varepsilon_R})}{[\mathrm{Re}(\sqrt{\varepsilon_R})+1]^2}\exp\left[-\frac{2\mathrm{Im}(\sqrt{\varepsilon_R})\omega d}{c}\right], \varphi_R = \beta_R d \end{aligned} \tag{S7}$$

where d = 500 μm is the thickness of the InSb layer. As shown in Fig. S1, the transmittance (in dB) maps for the R and L spin states are observed. It shows a strong gyro-mirror symmetry for these two orthogonal states: Fig. S2(a) shows the transmittance map of R spin state, with the increasing of the positive MF, the f_c (over the white region with the transmittance <-30dB) moves to the lower frequencies, but for the negative MF, the forbidden region moves to the higher frequencies. For L spin state propagating backward, when the MF direction is unchanged relative to the absolute coordinate system, the MF has reversed relative to the backward propagation direction, which is equivalent to the result that the forward transmission with the negative MF in Fig. S2(a). Therefore, the longitudinally magnetized InSb shows the

nonreciprocal one-way transmission for the R or L spin state in the THz regime or called nonreciprocal circular dichroism. However, for the L spin state, the transmittance map is reversed with that of the R spin state to the MF or propagation direction as shown in Fig. S2(b), showing the gyro-mirror symmetry.

Fig. S2(c)-S2(f) shows the experimental results of the magneto-optical effect of InSb in the THz band. As shown in Fig. S2(d) and S2(e), in the frequency range of 0.2~1 THz, the bulk InSb shows a high pass filtering characteristic with a cutting frequency f_c. As the MF changes from the negative to the positive value, the f_c of the R state moves to a lower frequency, while the f_c of the L state shifts to a higher frequency. If the direction of the MF is unchanged and the propagation direction is changed, both R and L states can only transmit in one direction, so the longitudinally magnetized InSb has nonreciprocity. The isolation band ranges from 0.25~0.75 THz, of which bandwidth is 500 GHz and the maximum value is 28 dB. It is also noted that the spectral lines of R and L states are completely mirror-symmetric to the MF or propagation direction.

For the same magnetic field, the difference between the transmittance of R and L is called circular dichroism (CD), and the difference between the phase angles is called optical activity (OA), which is essentially the Faraday rotation (FR) in this work. Both CD and FR reflect the chiral response of the sample. The experimental CD and FR spectra are also shown in Figs. S2(c) and S2(f), respectively. When the $B = 0$ T, the InSb has no chiral response. When the MF is applied, the InSb obtained the chiral response, making the CD and FR increase with the MFs. In the cyclotron resonance band, InSb mainly exhibits strong magnetic circular dichroism, while in the remote cyclotron resonance band, InSb exhibits the Faraday rotation effect. When $B > 0$, $A_R > A_L$, the CD and FR angle > 0, and when $B < 0$, the CD and FR angle < 0, which means that they are antisymmetric to the MFs. This phenomenon arises from the conjugate symmetry between R and L spin states. Thus, we can conclude: (1) the InSb shows the *chirality* for the same direction of MF; (2) InSb has the *nonreciprocity* for a certain spin state; (3) the chirality and isolation for the two conjugate spin states in the InSb are mirror-symmetric to the MF, that is to say, it has a *spin-conjugate symmetry*. All the relations between the spin states in the InSb can be expressed as follows:

$$\begin{aligned}&\text{Optical Chirality: } t_{R+} \neq t_{L+},\ t_{R-} \neq t_{L-};\\&\text{Nonreciprocity: } t_{R+} \neq t_{R-},\ t_{L+} \neq t_{L-};\\&\text{Conjugate symmetry: } t_{R+} = t_{L-},\ t_{R-} = t_{L+}\end{aligned} \tag{S8}$$

S3. Anisotropic optical response of monolayer metasurface

For the one layer of the anisotropic metasurface, the transfer matrix can be written as:

$$T_{MS}(\theta)=\begin{bmatrix} S_1 & \Delta_{MS} \\ \Delta_{MS} & S_2 \end{bmatrix},\begin{cases} S_1 = A_x\cos^2\theta + A_y e^{i\varphi}\sin^2\theta \\ S_2 = A_x\sin^2\theta + A_y e^{i\varphi}\cos^2\theta \\ \Delta_{MS} = A_x\sin\theta\cos\theta - A_y e^{i\varphi}\sin\theta\cos\theta \end{cases}, \quad \text{(S9)}$$

where θ is the rotational angle of the main axis, A_x and A_y denote the transmittance for the x-LP and y-LP components, and φ is the phase difference between these two LP components. The design of this single-layer anisotropic metasurface should meet the following requirements to obtain a sufficiently broadband nonreciprocal chiral response:

1) The metasurface should have a significant uniaxial anisotropic response (*i.e.* $t_x \neq t_y$ for x- and y- LP responses) in the high-frequency band which coincides with the Faraday rotation effect band of InSb); 2) The metasurface is isotropic (*i.e.* $t_x = t_y$ for x- and y- LP responses) in the low-frequency band which coincides with the cyclotron resonance band of InSb). After optimization, the double-L-shaped metallic can realize the demand as shown in Fig. 1(c) in the main text.

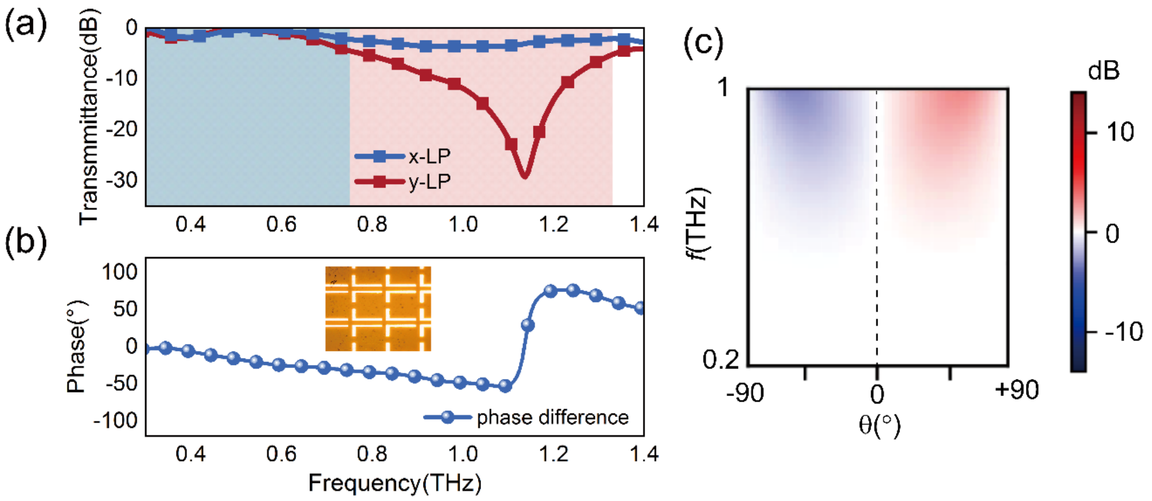

Fig. S3. The experimental (a) transmittance and (b) phase for the monolayer metasurface. (c) The intrinsic chirality characterized by CD for the MOMM when $B = 0$ T

The experiment transmission and phase of this metasurface can be found in Figs. S3(a) and S3(b), respectively. When the frequency f < 0.75THz (blue dashed region in Fig. S3(a)), the intensity transmittance of the x-LP component (A_x^2) is almost equal to the transmittance of the y-LP component (A_y^2), and the phase difference φ is less than 30°, in which case the metasurface

can be treated as the isotropic medium. However, when $f > 0.75$THz (pink dashed region in Fig. S3(a)), the transmittance and phase between the *x*-LP component and *y*-LP component enlarge, so this metasurface is an anisotropic medium.

S4. The theoretical calculation for MOMM and conjugate symmetry breaking

Next, we discuss the transfer matrix of MOMM. The transfer matrix of InSb under the LP basis vectors transformed from the CP basis vectors in Eq. S6 is:

$$T_{InSb} = \begin{bmatrix} S & -i\Delta_{InSb} \\ i\Delta_{InSb} & S \end{bmatrix} = \frac{1}{2}\begin{bmatrix} A_R e^{i\varphi_R} + A_L e^{i\varphi_L} & -i(A_R e^{i\varphi_R} - A_L e^{i\varphi_L}) \\ i(A_R e^{i\varphi_R} - A_L e^{i\varphi_L}) & A_R e^{i\varphi_R} + A_L e^{i\varphi_L} \end{bmatrix}$$

where A_R and A_L denote the amplitude transmittance of L and R state for InSb, φ_R and φ_L denote the phase of L and R state for InSb described in Eq. S6. Then, set the rotated angle of the first metasurface is 0, and the second one is θ, we can obtain the total transfer matrix as:

$$T_{all-LP} = T_{MS1}(0)T_{InSb}T_{MS2}(\theta) = \begin{bmatrix} A_x(S_1 S + i\Delta_{MS}\Delta_{InSb}) & A_y e^{i\varphi}(-iS_1\Delta_{InSb} + \Delta_{MS}S) \\ A_x(\Delta_{MS}S + iS_2\Delta_{InSb}) & A_y e^{i\varphi}(-i\Delta_{MS}\Delta_{InSb} + SS_2) \end{bmatrix}, \quad \text{(S10)}$$

To more clearly identify the transmission properties of the MOMM, the above equation can be transferred to a matrix in the CP basis vectors:

$$\begin{bmatrix} E_x \\ E_y \end{bmatrix} = \frac{1}{\sqrt{2}}\begin{bmatrix} 1 & 1 \\ i & -i \end{bmatrix}\begin{bmatrix} E_R \\ E_L \end{bmatrix} = C\begin{bmatrix} E_R \\ E_L \end{bmatrix},$$

Thus, the total transmission matrix on the CP basis can be written as:

$$T_{all-CP} = \begin{bmatrix} T_{RR} & T_{RL} \\ T_{LR} & T_{LL} \end{bmatrix} = C^{-1}T_{all-LP}C$$
$$= \frac{1}{2}\begin{bmatrix} 1 & -i \\ 1 & i \end{bmatrix}\begin{bmatrix} A_x(S_1 S + i\Delta_{MS}\Delta_{InSb}) & A_y e^{i\varphi}(-iS_1\Delta_{InSb} + \Delta_{MS}S) \\ A_x(\Delta_{MS}S + iS_2\Delta_{InSb}) & A_y e^{i\varphi}(-i\Delta_{MS}\Delta_{InSb} + SS_2) \end{bmatrix}\begin{bmatrix} 1 & 1 \\ i & -i \end{bmatrix}$$
$$= \frac{1}{4}\begin{bmatrix} (A_x + A_y e^{i\varphi})^2 A_R e^{i\varphi_R} + (A_x - A_y e^{i\varphi})^2 A_L e^{i(\varphi_L - 2\theta)} & (A_x^2 - A_y^2 e^{i2\varphi})(A_R e^{i\varphi_R} + A_L e^{i(\varphi_L - 2\theta)}) \\ (A_x^2 - A_y^2 e^{i2\varphi})(A_R e^{i\varphi_R} + A_L e^{i(\varphi_L + 2\theta)}) & (A_x + A_y e^{i\varphi})^2 A_L e^{i\varphi_L} + (A_x - A_y e^{i\varphi})^2 A_R e^{i(\varphi_R + 2\theta)} \end{bmatrix}$$

(S11)

The difference between the two spin-flip states T_{RL} and T_{LR} are only dependent on the structures of the moiré metasurface, with no relation to the InSb. In this work, we have designed the geometry of the moiré metasurface as shown in Fig. 1 in the main text, which makes the spin-flip states T_{RL} and T_{LR} (*i.e.* the spin conversion) to be negligible, so we're only interested in the spin-locked states in all the discussions, and we simplify the spin-locked states T_{RR} and T_{LL} to T_R and T_L, respectively.

Next, we discuss a few important cases:

1) When there is no MF applied $B = 0$, InSb is an isotropic medium in this case, $A_R = A_L = A_0$ and $\varphi_R = \varphi_L = \varphi_0$, Eq. S10 simplifies as follows:

$$\begin{cases} T_R = \dfrac{(A_x + A_y e^{i\varphi})^2 A_0 e^{i\varphi_0} + (A_x - A_y e^{i\varphi})^2 A_0 e^{i(\varphi_0 - 2\theta)}}{4} \\ T_L = \dfrac{(A_x + A_y e^{i\varphi})^2 A_0 e^{i\varphi_0} + (A_x - A_y e^{i\varphi})^2 A_0 e^{i(\varphi_0 + 2\theta)}}{4} \end{cases} \quad \text{(S12)}$$

Thus, we calculate the CD map as a function of frequency and the rotational angle as shown in Fig. S3(c). As we can see, when the twisted angle $\theta \neq 0°$ or 90°, the CD map is not 0 at a higher frequency which means $T_R \neq T_L$. As a result of the spatial mirror-symmetry breaking, the MOMM has an intrinsic chirality. The CD map is mirror symmetric with the rotational angle: $T_R > T_L$ for positive angles and $T_R < T_L$ for negative angles. However, this chiral transmission for the spin states is reciprocal due to the time-reversal symmetry. This is a *reciprocal chirality*.

2) When the MF is applied $B \neq 0$, for InSb, $A_R \neq A_L$ and $\varphi_R \neq \varphi_L$, the chirality and nonreciprocity of MOMM can be influenced by the InSb under the different MFs. When $\theta = 0°$,

$$\begin{cases} T_R = \dfrac{(A_x + A_y e^{i\varphi})^2 t_R + (A_x - A_y e^{i\varphi})^2 t_L}{4} \\ T_L = \dfrac{(A_x + A_y e^{i\varphi})^2 t_L + (A_x - A_y e^{i\varphi})^2 t_R}{4} \end{cases}, \quad \text{(S13)}$$

When $\theta = 90°$,

$$\begin{cases} T_R = \dfrac{(A_x + A_y e^{i\varphi})^2 t_R - (A_x - A_y e^{i\varphi})^2 t_L}{4} \\ T_L = \dfrac{(A_x + A_y e^{i\varphi})^2 t_L - (A_x - A_y e^{i\varphi})^2 t_R}{4} \end{cases}, \quad \text{(S14)}$$

When the direction of the MF (or the direction of propagation) is opposite, $T_{R+} = T_{L-}$ and $T_{R-} = T_{L+}$. The same conclusion is obtained as Eq. S7. The MOMM exhibits a *nonreciprocal chirality with spin-conjugate symmetry*.

3) When $B \neq 0$, $\theta \neq 0°$, ±90°, and 180°, the relations between the spin states through MOMM can be expressed by Eq. 3 in the main text as follows:

Optical Chirality: $T_{R+} \neq T_{L+}$, $T_{R-} \neq T_{L-}$;
Nonreciprocity: $T_{R+} \neq T_{R-}$, $T_{L+} \neq T_{L-}$;
Conjugate Asymmetry: $T_{R+} \neq T_{L-}$, $T_{R-} \neq T_{L+}$

This MOMM exhibits a *nonreciprocal chirality with spin-conjugate symmetry breaking.*

If we suppose $T_R = 0$,

$$\begin{aligned} &T_{R+} = \frac{1}{4}(A_x + A_y e^{i\varphi})^2 t_{R+} + \frac{1}{4}(A_x - A_y e^{i\varphi})^2 t_{L+} e^{i(-2\theta)} = 0 \\ &\Rightarrow -\frac{t_{L+}}{t_{R+}}\left(\frac{A_x - A_y e^{i\varphi}}{A_x + A_y e^{i\varphi}}\right)^2 = e^{i2\theta}, \end{aligned} \tag{S15}$$

Here, $T_{L+} = A_x A_y e^{i\varphi}(t_{L+} - t_{R+}e^{i2\theta})$. However, when the MF is reversed, $t_{R-} = t_{L+}$ and $t_{L-} = t_{R+}$ in Eq. S8, and

$$\begin{aligned} T_{R-} &= \frac{1}{4}(t_x + t_y e^{i\varphi})^2 t_{L+} + \frac{1}{4}(t_x - t_y e^{i\varphi})^2 t_{R+} e^{i(-2\theta)} \\ T_{L-} &= \frac{1}{4}(A_x + A_y e^{i\varphi})^2 t_{R+} + \frac{1}{4}(A_x - A_y e^{i\varphi})^2 t_{L+} e^{i(2\theta)} = \frac{i}{2}(A_x - A_y e^{i\varphi})^2 t_{L+}(\sin 2\theta) \end{aligned}$$

In summary, when Eq. S15 is satisfied,

$$\begin{cases} T_{R+} = 0 \\ T_{L+} = A_x A_y e^{i\varphi}(t_{L+} - t_{R+}e^{i2\theta_{\mathrm{M}}}) \\ T_{R-} = \frac{1}{4}(A_x + A_y e^{i\varphi})^2 t_{L-} + \frac{1}{4}(A_x - A_y e^{i\varphi})^2 t_{R-} e^{i(-2\theta_{\mathrm{M}})} \\ T_{L-} = \frac{i}{2}(A_x - A_y e^{i\varphi})^2 t_{L-}(\sin 2\theta_{\mathrm{M}}) \end{cases} \tag{S16}$$

The analysis of the condition on the conjugate symmetry breaking in Eq. S15 (Eq. 4 in the main text) and moiré angle θ_{M} in Eq. S16 (Eq. 5 in the main text) has been described in detail in the main text.

4) In the third case above, there is a special case. In the cyclotron resonance band of InSb (in the band of 0.24-0.75 THz at B = 0.17 T) in Sec. S2, when the MF is positive, $A_L = 0$,

$$T_{all-CP+} = \frac{1}{4}\begin{bmatrix} (A_x + A_y e^{i\varphi})^2 A_R\, \mathrm{e}^{i\varphi_R} & (A_x^2 - A_y^2 e^{i2\varphi})A_R\, \mathrm{e}^{i\varphi_R} \\ (A_x^2 - A_y^2 e^{i2\varphi})A_R\, \mathrm{e}^{i\varphi_R + 2\theta} & (A_x - A_y e^{i\varphi})^2 A_R\, \mathrm{e}^{i\varphi_R + 2\theta} \end{bmatrix}$$

$$T_{R+} = (A_x + A_y e^{i\varphi})^2 A_R\, \mathrm{e}^{i\varphi_R}/4, \quad T_{L+} = (A_x - A_y e^{i\varphi})^2 A_R\, \mathrm{e}^{i\varphi_R + 2\theta}/4$$

When the MF is reversed, $A_R = 0$,

$$T_{all-CP-} = \frac{1}{4}\begin{bmatrix} (A_x - A_y e^{i\varphi})^2 A_L \, \mathrm{e}^{i\varphi_L - 2\theta} & (A_x^{\,2} - A_y^{\,2} e^{i2\varphi}) A_L \, \mathrm{e}^{i\varphi_L - 2\theta} \\ (A_x^{\,2} - A_y^{\,2} e^{i2\varphi}) A_L \, \mathrm{e}^{i\varphi_L} & (A_x + A_y e^{i\varphi})^2 A_L \, \mathrm{e}^{i\varphi_L} \end{bmatrix}$$

$$T_{R-} = (A_x - A_y e^{i\varphi})^2 A_L \, \mathrm{e}^{i\varphi_L - 2\theta} / 4, \quad T_{L-} = (A_x + A_y e^{i\varphi})^2 A_L \, \mathrm{e}^{i\varphi_L} / 4$$

Moreover, as discussed in Sec. S3, when $f < 0.75$THz, the monolayer metasurface can be treated as an isotropic medium. Thus, $A_x – A_y = 0$, in this case, Eq. S16 can be changed as:

$$\begin{cases} T_{R+} = (A_x + A_y e^{i\varphi})^2 A_R \, \mathrm{e}^{i\varphi_R} / 4 \\ T_{L+} = 0 \\ T_{R-} = 0 \\ T_{L-} = (A_x + A_y e^{i\varphi})^2 A_L \, \mathrm{e}^{i\varphi_L} / 4 \end{cases} \tag{S17}$$

In this case, the MOMM turns to a *nonreciprocal chirality with spin-conjugate symmetry*.

In summary, by combining the InSb with the moiré metasurfaces, in both cyclotron resonance and Faraday band, we can get the nonreciprocity and chirality, enhancing the isolation and CD effect. And in the Faraday effect band, a unique property of spin-conjugate symmetry breaking can be achieved.

S5. Superchiral field

An object has chirality when its mirror image is not identical to itself. For optical chirality, it means a chiral object has different absorption cross sections when illuminated with LCP or RCP light. This different absorption is measured by the dissymmetry factor g which is defined as $g = 2(a_L - a_R)/(a_L + a_R)$, where $a_{L(R)}$ is the absorption rate in LCP or RCP light. However, for most natural material molecules, $g < 10^{-3}$, thus the concept of the *superchiral field* has been introduced that displays greater chiral asymmetry than that of the CP plane wave. It is demonstrated that by applying the superchiral field, the sensitivity of chiroptical measurement could be greatly enhanced. The superchiral field can be characterized by the following time-even pseudoscalar, termed the optical chirality:

$$C \equiv -\frac{\omega\varepsilon_0}{2}\mathrm{Im}(E^* \bullet B) \tag{S18}$$

where E and B are the local electric and magnetic fields, and E^* denotes the complex conjugate of the electric field. For the common R or L spin state in the free space, the chirality $C_{CPL} = \pm\omega\varepsilon_0|E_0|^2/2c$, where $c = 3\times10^8$ m/s is the speed of light in the vacuum. However, the localization of the light field or the complex interference of light can occur super chirality. In this condition, the optical chirality is enhanced by a chirality enhancement index S_χ defined as:

$$S_\chi \equiv \frac{C}{|C_{CPL}|} = -\frac{c\,\mathrm{Im}\left[\tilde{E}^* \bullet \tilde{B}\right]}{|E_{CPL}|^2} \tag{S19}$$

The chirality enhancement index S_χ of the MOMM changes the chiral response of both InSb and moiré metasurface themselves. And the sign indicates the different chiral spin states (L or R). Therefore, since the superchiral field is asymmetric or not identical as shown in Fig. 3(c) in the main text, the chirality of MOMM is different in different distribution and enhancement features for the different incident spin states under the positive or negative MFs.

S6. Simulation results of the MOMM

Here, we simulate the transmittance for the MOMM with different magnetic fields and twisted angles, which is correspond to Fig. 5. Due to the twisted angle between two layers of the metallic metasurfaces, the period of original metasurfaces will not applicable for the moiré structure. For simplicity, we use an equivalent parameter method to restrict the simulation region to a unit period. When $\theta = 0°$ as shown in Fig. S4(a, d) and $\theta = 90°$ as shown in Fig. S4(c, f), a nonreciprocal transmission is exhibited, but for L and R states, their spectra are mirror-symmetric to the MFs. When $\theta = 45°$, it also has non-reciprocity as shown in Fig. S4(b, e). Moreover, the spectra between the L and R states are asymmetric to the MFs, which indicates the spin-conjugate symmetry breaking in the higher frequency band.

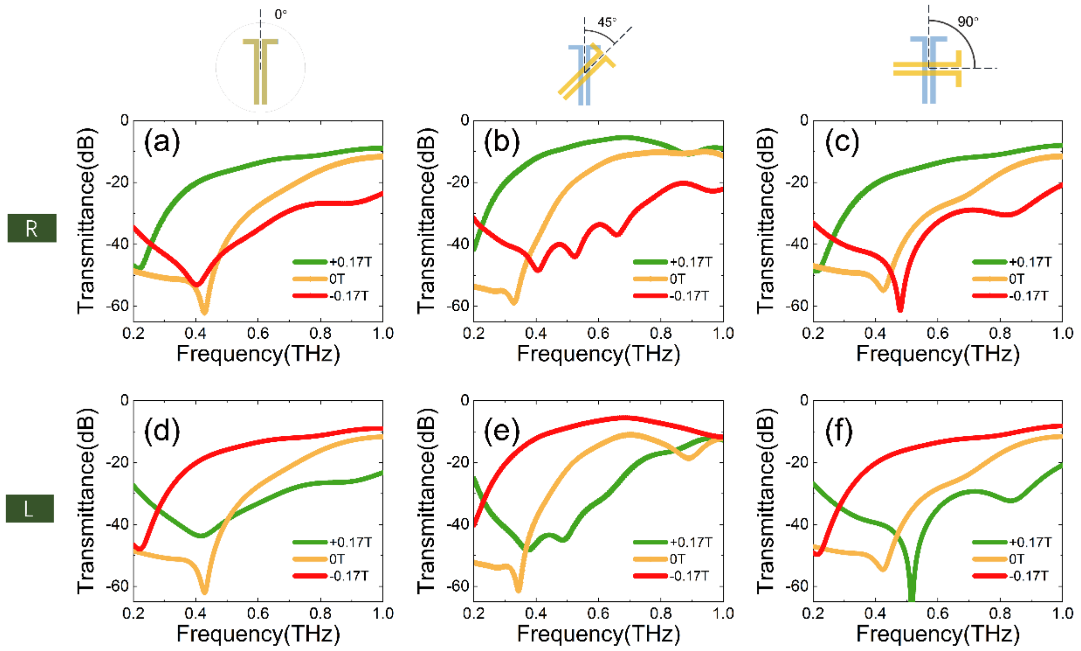

Fig. S4. The simulative transmission spectra of different MOMM for the different spin states under several MFs: T_R with θ= (a) 0°, (b) 45°, and (c) 90°; T_L with θ= (d) 0°, (e) 45°, and (f) 90°.

References

[1] P. C. M. Planken, H.-K. Nienhuys, H. J. Bakker, T. Wenckebach, *J. Opt. Soc. Am. B* **2001**, *18* (3), 313, https://doi.org/10.1364/JOSAB.18.000313.

[2] Q. Mu, F. Fan, S. Chen, S. Xu, C. Xiong, X. Zhang, X. Wang, S. Chang, *Photon. Res.* **2019**, *7* (3), 325, https://doi.org/10.1364/PRJ.7.000325.

[3] X. Wang, A. A. Belyanin, S. A. Crooker, D. M. Mittleman, J. Kono, *Nature Physics* **2010**, *6* (2), 126, https://doi.org/10.1038/nphys1480.

[4] S. Lin, S. Silva, J. Zhou, D. Talbayev, *Advanced Optical Materials* **2018**, *6* (19), 1800572, https://doi.org/https://doi.org/10.1002/adom.201800572.

[5] M. Oszwałldowski, M. Zimpel, *Journal of Physics and Chemistry of Solids* **1988**, *49* (10), 1179, https://doi.org/https://doi.org/10.1016/0022-3697(88)90173-4.

[6] B. He, J. Fan, Y. Cheng, F. Chen, H. Luo, R. Gong, *J. Opt. Soc. Am. B* **2021**, 38 (5), 1518, https://doi.org/10.1364/JOSAB.420928.

[7] F. Chen, Y. Cheng, H. Luo, *IEEE Access* **2020**, 8, 82981, https://doi.org/10.1109/ACCESS.2020.2991331.
